# Metabolic and Target-Site Mechanisms Combine to Confer Strong DDT Resistance in *Anopheles gambiae*

**DOI:** 10.1371/journal.pone.0092662

**Published:** 2014-03-27

**Authors:** Sara N. Mitchell, Daniel J. Rigden, Andrew J. Dowd, Fang Lu, Craig S. Wilding, David Weetman, Samuel Dadzie, Adam M. Jenkins, Kimberly Regna, Pelagie Boko, Luc Djogbenou, Marc A. T. Muskavitch, Hilary Ranson, Mark J. I. Paine, Olga Mayans, Martin J. Donnelly

**Affiliations:** 1 Department of Vector Biology, Liverpool School of Tropical Medicine, Liverpool, United Kingdom; 2 Institute of Integrative Biology, University of Liverpool, Liverpool, United Kingdom; 3 Noguchi Memorial Institute for Medical Research, University of Ghana, Legon, Ghana; 4 Boston College, Chestnut Hill, Massachusetts, United States of America; 5 Institut Régional de Santé Publique de Ouidah/Université d’Abomey-Calavi, Cotonou, Bénin; 6 Harvard School of Public Health, Boston, Massachusetts, United States of America; 7 Malaria Programme, Wellcome Trust Sanger Institute, Hinxton, Cambridge, United Kingdom; Kansas State University, United States of America

## Abstract

The development of resistance to insecticides has become a classic exemplar of evolution occurring within human time scales. In this study we demonstrate how resistance to DDT in the major African malaria vector *Anopheles gambiae* is a result of both target-site resistance mechanisms that have introgressed between incipient species (the M- and S-molecular forms) and allelic variants in a DDT-detoxifying enzyme. Sequencing of the detoxification enzyme, *Gste2*, from DDT resistant and susceptible strains of *An. gambiae*, revealed a non-synonymous polymorphism (I114T), proximal to the DDT binding domain, which segregated with strain phenotype. Recombinant protein expression and DDT metabolism analysis revealed that the proteins from the susceptible strain lost activity at higher DDT concentrations, characteristic of substrate inhibition. The effect of I114T on GSTE2 protein structure was explored through X-ray crystallography. The amino acid exchange in the DDT-resistant strain introduced a hydroxyl group nearby the hydrophobic DDT-binding region. The exchange does not result in structural alterations but is predicted to facilitate local dynamics and enzyme activity. Expression of both wild-type and 114T alleles the allele in *Drosophila* conferred an increase in DDT tolerance. The 114T mutation was significantly associated with DDT resistance in wild caught M-form populations and acts in concert with target-site mutations in the voltage gated sodium channel (*Vgsc-1575Y* and *Vgsc-1014F*) to confer extreme levels of DDT resistance in wild caught *An. gambiae.*

## Introduction

Physiological resistance to insecticides often involves either mutations in the insecticide target site (target-site resistance), or elevated activity of detoxifying enzymes that metabolise and/or sequester insecticides (metabolic resistance). Resistance may result from selection upon standing genetic variation [1] or from a *de novo* mutation [2]. In *Anopheles gambiae*, a primary African malaria vector, a third route has been described, involving introgression of resistance mutation-bearing haplotypes between molecular forms which are thought to be in the process of speciation [3]. There is overwhelming evidence that the mutation *L1014F*, a replacement change in the voltage-gated sodium channel (*Vgsc*), the target of both DDT and pyrethroid insecticides, is significantly associated with increased phenotypic resistance in both the donor S- and recipient M- form populations across Africa [4], [5], [6]. However, what remains unknown is whether such introgressed resistance alleles interact with allelic variants in the recipient genetic background.

In *An. gambiae* metabolic resistance has been linked to elevated expression of detoxifying enzymes through microarray-based analyses and quantitative PCR [7], [8], [9]. An epsilon-class glutathione-S-transferase in *An. gambiae*, GSTE2, and its orthologue in the dengue and yellow fever vector *Aedes aegypti*, have been linked to DDT resistance through elevated gene expression [10], [11]. Recombinant protein expression and *in vitro* assays also support a role for this enzyme in DDT metabolism [11], [12]. In previous studies, *Gste2* was found to be 5–8 fold over-expressed in *An. gambiae* of the ZAN/U strain, which displays DDT resistance in the absence of mutations in the voltage-gated sodium channel, compared to a susceptible East African mosquito colony (Kisumu) [12], [13], [14].

The rationale for the current study arose from the serendipitous discovery of allelic differences in *Gste2* in recently re-established colonies of Kisumu and ZAN/U (source www.MR4.org), which exhibited the expected DDT susceptibility/resistance profiles but not the level of differential expression observed previously [12], [13]. The ZAN/U colony showed only a 2.34-fold greater expression of *Gste2* and less than a 2-fold difference in protein expression compared with the Kisumu colony (Figure S1 and Figure S2 in File S1). Upon review of the crystal structure that was already resolved for GSTE2 from the susceptible Kisumu strain [15], it appeared that the alleles differed in codons proximal to the putative DDT-binding site, a hydrophobic pocket adjacent to the glutathione (GSH) binding site.

Our study demonstrates how one substitution (I114T) is found commonly, and inferred to originate, in M-molecular form populations of *An. gambiae* where it is significantly associated with DDT resistance. In concert with target-site resistance mechanisms (*Vgsc-1014F* and *Vgsc*-*1575Y*), it explains a substantial fraction of the observed variation in DDT resistance. Recombinant protein expression, X-ray crystallography and transgenic expression of allelic variants in *Drosophila* are also presented to provide a mechanistic insight.

## Results

### Recombinant protein expression and DDTase activity screens

**Figure 1:**
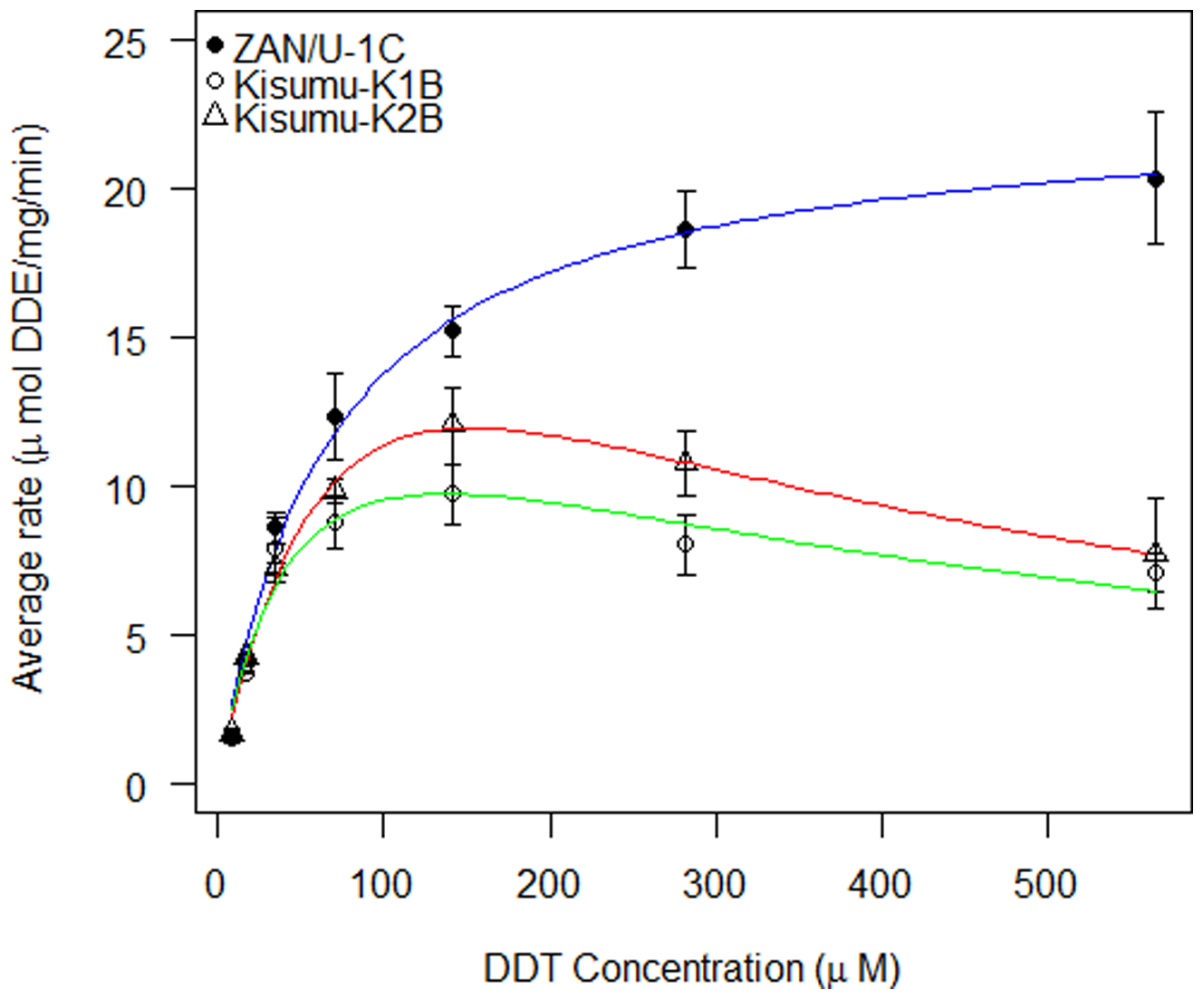
Comparison of GSTE2 catalysed DDT metabolism for three variant recombinant proteins over a DDT dilution series. Three allelic variants of enzyme GSTE2 from *An. gambiae* are compared over a range of DDT concentrations and the mean production of DDE plotted from three replicate assays. Fitted curves used the Michaelis-Menten equation for the ZAN/U allele and a substrate inhibition equation for the two Kisumu alleles

Based upon amino acid sequence, three allelic variants were identified, two within the Kisumu colony and one in the ZAN/U colony (Table 1; GenBank accession numbers: JX840597- JX840599). The three alleles were expressed in *E.coli* and each exhibited activity with the substrate CDNB in the presence of GSH; confirming that the expressed proteins were glutathione-S-transferases (Table 1). DDT metabolism assays were performed to determine optimal conditions for kinetic analysis of each variant *GSTE2* enzyme with a substrate (DDT) dilution series. At lower concentrations all three variant enzymes displayed comparable activity (Figure 1). However, the ZAN/U-derived *GSTE2* protein displayed a significantly higher mean enzyme rate than the two Kisumu proteins at the higher concentrations tested (Figure 1). Enzyme kinetic measurements did not produce markedly different values for maximum enzyme rate (V_max_) and the K_M_ (substrate concentration at half maximum velocity) for the three variants (Table 2). However, the Kisumu alleles did not exhibit standard Michaelis-Menten kinetics (Figure 1), but rather displayed profiles typical of enzymes experiencing substrate inhibition [16], [17].

**Table 1:** GSTE2 allelic variants from the *An. gambiae* Kisumu and ZAN/U strain used for recombinant protein expression.

Cloned variant	Amino acid position		Specific activity (μmoles/mg)
	114	120	
Kisumu 1B	Isoleucine	Leucine	15.85
Kisumu 2B	Isoleucine	Phenylalanine	21.33
ZAN/U 1C	Threonine	Phenylalanine	7.10

The position of variant amino acids proximal to the putative DDT binding site are shown; together with the specific activity of the recombinant GSTE2 with substrate CDNB. Protein concentrations were determined using a commercial assay (Fluka – Sigma-Aldrich) based on Bradford assay chemistry[40]. CDNB activity was determined by colorimetric assay and spectrophotometric reading.

**Table 2:** Enzyme kinetic parameters of three GSTE2 alleles with substrate DDT.

	Kisumu 1B	Kisumu 2B	ZAN/U 1C
*K_M_* ^DDT^(μM)	50.9	97.8	66.4
*V_max_* (μmol DDE/min/mg)	17.0	27.2	22.9
*K_cat_* (s^−1^)	14.1	22.5	18.9

Three variant GSTE2 proteins were expressed from a DDT resistant (ZAN/U) and susceptible (Kisumu) strain of *An. gambiae* and assayed with substrate DDT over a range of concentrations. The maximum enzyme rate (V_max_), substrate concentration at half the maximum rate (K_M_) and catalytic turn-over (K_cat_) were calculated for each protein from a Michaelis-Menten or substrate inhibition equation (Figure 1).

### Structural analysis of non-synonymous changes in *GSTE2*

Molecular modelling was used initially to investigate the mechanistic effect of the amino acid replacements on catalysis. Previously, Wang et al. [15] proposed that a hydrophobic pocket in close proximity to the GSH binding site was the site of DDT binding. Predicted to be of particular importance was the inclination of the C-terminal section of helix H4, which brought residues 112, 116 and 120 closer to the GSH cofactor. These residues also helped to form a pocket ‘cap’ for the putative DDT binding site, which would potentially increase hydrophobicity and therefore affinity for the highly hydrophobic DDT molecule. Our study focused upon two residue exchanges, I114T and F120L, which are located in the C-terminal section of helix H4 and, thus, have the potential to influence DDT binding.

The variable mutation found at position 120, F120L, in the Kisumu strain had potential to affect the formation of the putative DDT pocket cap as the aromatic phenyalanine is replaced with the shorter aliphatic chain of leucine. F120 is predicted to make hydrophobic contact with one of the aromatic rings of the DDT molecule. A leucine residue at this position, being smaller, may not form as tight an interaction with the DDT and, thereby, weaken its binding. The importance of the phenylalanine residue at this position is supported by the likelihood that this is the ancestral allele, as it is fixed in an extensive collection of *An. arabiensis* from Sudan, Ethiopia, Tanzania and Malawi (collection details in [18] (GenBank accession numbers: JX627247-JX627266). However, enzyme kinetics parameters (Table 2) indicate that the F120L exchange has little influence on substrate affinity or catalysis, suggesting that the aromatic group of phenylalanine is dispensable at this position and not deterministic of DDT affinity.

**Figure 2:**
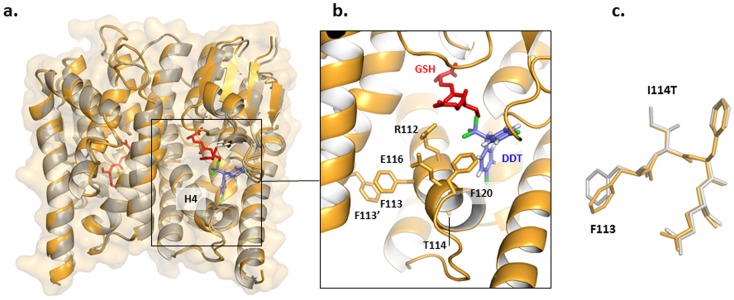
Crystal structure of *GSTE2* ZAN/U variant. a. Superposition of the crystal structure of ZAN/U determined in this study (orange) and the Kimusu 1B variant (grey; PDB entry IMI). A high degree of local and overall structural agreement is clearly noticeable. The location of the docked DDT is based on the computational prediction of Wang et al.[15]. Some manual adjustments were made to relieve steric clashes and to better superimpose the DDT on the position of the hexyl group of bound S-hexylglutathione. b. Close-up detail of the ZAN/U active site. c. Superposition of structure of ZAN/U and Kimusu 1B variant local to position 114 (colour code as in a. A superimposition of ZAN/U from *An. gambiae* with the *GSTE2* from *An. funestus* is provided in Figure S3).

**Figure 3:**
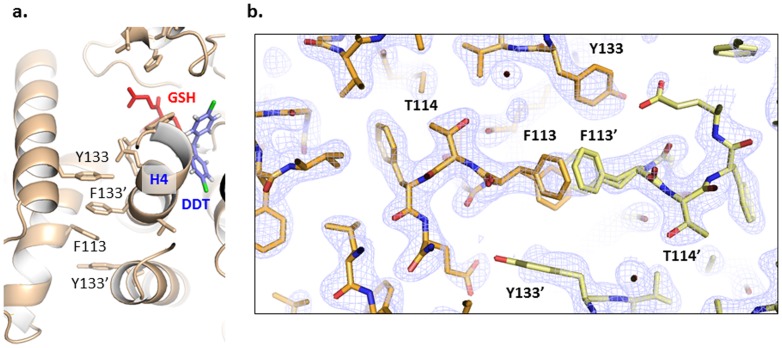
Subunit Interface in *GSTE2* variants. a. Close-up detail of interface groups in the *GSTE2* dimer. Phenylalanine residues F113 contributed by the respective helices H4 as well as tyrosines Y133 from neighbouring helices pack together to form a linear stack. b. Electron density map (contoured at 1.0 σ) for the *GSTE2* ZAN/U variant. The mutated residue T114 is shown. The preceding residue F113 is poorly ordered and has been modeled as adopting two alternate conformations (towards the front and back of the paper plane).

Position 114 is also situated in close proximity to the predicted DDT binding pocket. The effect of the change from isoleucine, inferred to be ancestral from comparisons with the same *An. arabiensis* data, to threonine at position 114 was difficult to estimate through modelling. In this case, a destabilizing polar hydroxyl group is introduced in a hydrophobic core region of the protein in ZAN/U, with the potential for marked effects on protein conformation. To better u*Nde*rstand the effect of this substitution in enzyme activation, we elucidated the structure of ZAN/U:GSH using X-ray crystallography (Figure 2). The structure, determined to 2.3 Å resolution (R-factor/R-free 17.57/22.78 %; Table S2 in File S1), closely resembles that of the Kisumu enzyme previously reported (PDB entry 2IMI; [15]) (0.5 Å overall rmsd calculated using RAPIDO [19] (Figure 2) as well as that of *GSTE2* from *An. funestus* most recently elucidated (PDB entry 3ZML). Similar to the Kisumu variant from *An. gambiae*, the latter carries Ile at position 114. Both enzymes share 93% sequence identity and their structures superimpose with an rmsd of 0.3 Å. The model of ZAN/U calculated in this study shows that the introduced hydroxyl group is stabilized by hydrogen bond formation to the main chain carbonyl group of R110 (calculated using HBOND, J. Overington, unpublished), so that the presence of this polar group in the hydrophobic core does not lead to structural alterations in the enzyme (Fig 2a; a comparison to *GSTE2* from *An. funestus* is shown in Figure S3 in File S1). Interestingly, inspection of electron density maps for all *GSTE2* enzymes (Figure 3), calculated using PDB_REDO [20], reveal a disorder of residues F113 and Y133, which are involved in the mutual packing of two H4 helices across the dimer interface, at a spot that is immediately local to the predicted DDT pocket. This suggests that this region, which constitutes the DDT pocket ‘cap’, has high intrinsic dynamics. Such dynamics could facilitate the motions that take place during substrate binding and/or product release and, thereby, influence catalytic turn-over. Our data suggest that mutations can influence catalysis even when not resulting in detectable structural alterations, most likely by affecting the molecular dynamics of this region.

### Heterologous expression of *GSTE2* in *Drosophila melanogaster*

**Figure 4:**
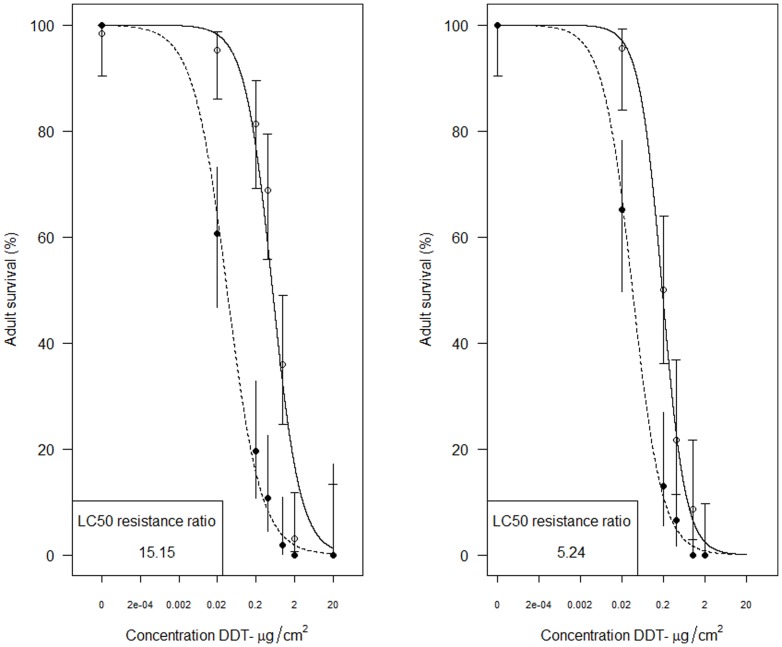
Dose-response curves for *Drosophila melanogaster* adults transformed with *Anopheles gambiae Gste2* alleles. The left panel shows survival of control (CyO x UAS+*Gste2*-Kisumu1B; black circles) and Kisumu allele expressing lines (*Actin*-Gal4 x UAS+*Gste2*-Kisumu1B; open circle) together with 95% confidence intervals. The right panel shows survival of control (CyO x UAS+*Gste2*-ZANU; black circles) and ZAN/U allele (Actin-Gal4 x UAS+*Gste2*-ZANU; open circle) together with 95% confidence intervals.

Heterologous expression in *Drosophila melanogaster* was achieved for both the *Gste2*-ZAN/U and *Gste2*-Kisumu1B alleles (Figure 4). For both alleles ubiquitous expression of *An. gambiae Gste2* resulted in an increase in resistance to DDT as assessed by resistance ratio of LC50s (LC50 transformed line/LC50 control). Although, contrary to the recombinant *E.coli* work (Figure 1), and our a priori expectations, the resistance ratios were apparently higher for *Gste2*-Kisumu1B (15.15) than *Gste2*-ZAN/U (5.24).

### Screening of I114T and *Vgsc* variants in wild-caught, DDT-phenotyped specimens of *An. gambiae*

**Figure 5:**
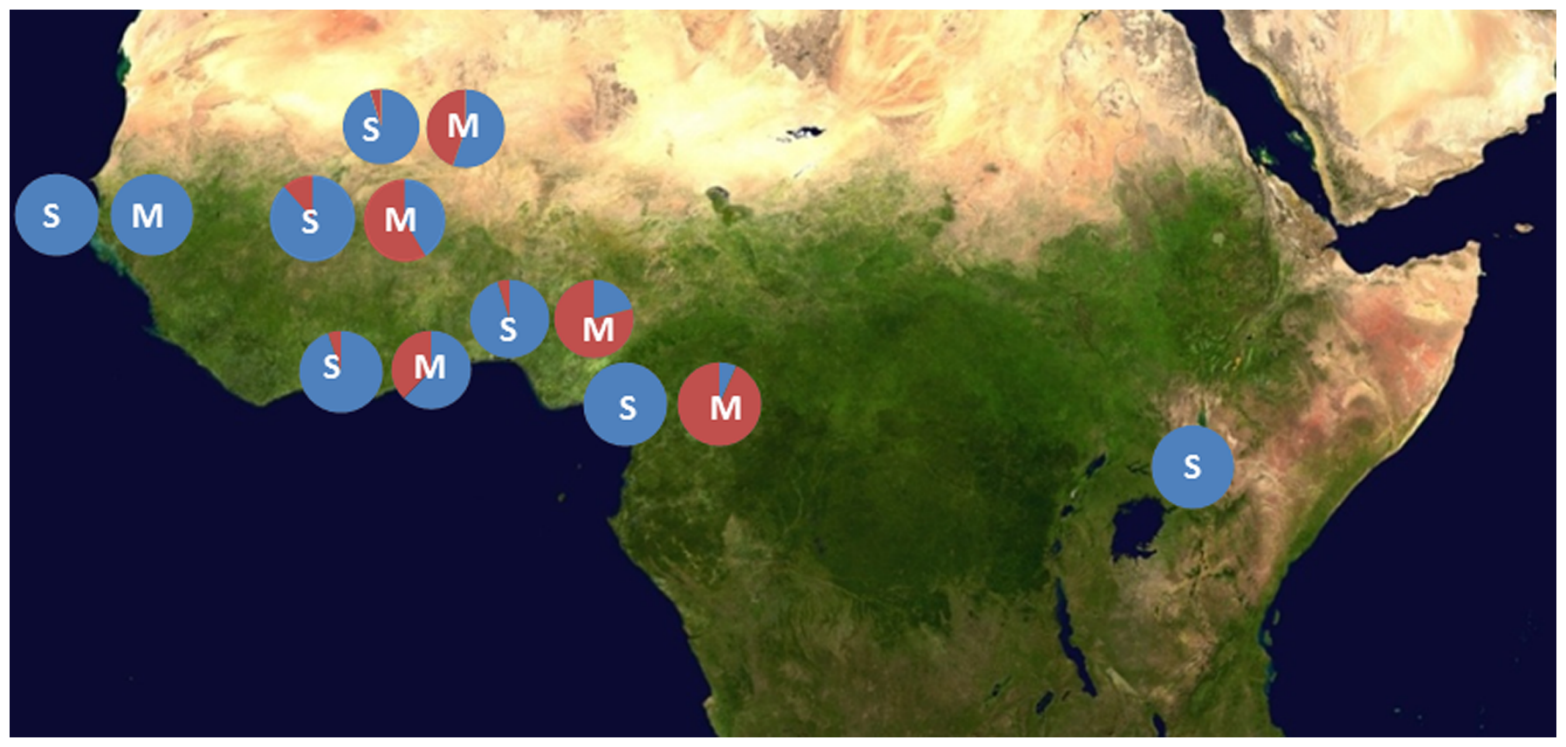
Geographical variation in frequency of *Gste2*-I114T in the S and M molecular forms of *An. gambiae* across Africa. Blue represents the I114 and red the T114 frequency. The molecular form of the collection is indicated by the letter overlaid on each chart. Samples were from: Benin S-form n = 111; M-form n = 223. Burkina Faso S-form n = 115; M-form n = 216. Cameroon S-form n = 55; M-form n = 652. Ghana S-form n = 29; M-form n = 758. Guinea-Bissau S-form n = 38; M-form n = 39. Mali S-form n = 31; M-form n = 26. Uganda S-form n = 207. The base map was obtained from http://en.wikipedia.org/wiki/File:Africa_satellite_orthographic.jpg and was created by NASA. Details of the locations are given in Table S3 in File S1.

We screened for the presence of the I114T mutations in a number of collections of both molecular forms of *An. gambiae*. Unexpectedly, given that the ZAN/U colony is of the S-molecular form and originates from East Africa, the 114T allele was most common in M-form populations from West Africa (Figure 5). For example in both Benin and Burkina Faso 114T allele was significantly more frequent in M-form (Benin Freq = 0.79; 95%CIs 0.75–0.83: Burkina Faso Freq = 0.59; 95%CIs 0.54–0.63) than sympatric S-form populations (Benin Freq =  0.05; 95%CIs 0.03–0.09: Burkina Faso Freq = 0.12; 95%CIs 0.08–0.17) suggestive that the mutation originated in M-form populations. Consequently we focused genotype: phenotype studies on West African populations, where in addition we were able to investigate potential interactions between the *Gste2* variant and two known DDT-linked *Vgsc* variants that are rare or absent in East Africa. Female *An. gambiae* from Benin and Burkina Faso that survived or were killed by 60 minute DDT exposure in standard WHO susceptibility tests[21], were genotyped at the *Gste2*-114 codon and at the resistance-associated mutations in the voltage gated sodium channel (*Vgsc-1014F*, commonly termed *kdr*, and *Vgsc* -1575Y) [5]. In the M-form specimens from Benin there was a significant association between 114T and DDT survival (allelic test of association p = 8×10–4: Odds Ratio(OR) = 2.35 ; 95%CIs 1.42–3.88).The trend was similar in Burkinabe specimens but did not reach statistical significance (p = 0.28: OR =  1.27; 95%CIs 0.83–1.93). As expected the *Vgsc-1014F* mutation was associated with DDT resistance in both locations, Benin (p = 6×10^−4^: OR =  2.21; 95%CIs 1.40–3.50) and Burkina Faso (p = 5×10^−7^; OR =  3.05 95%CIs 1.97–4.74).

**Figure 6:**
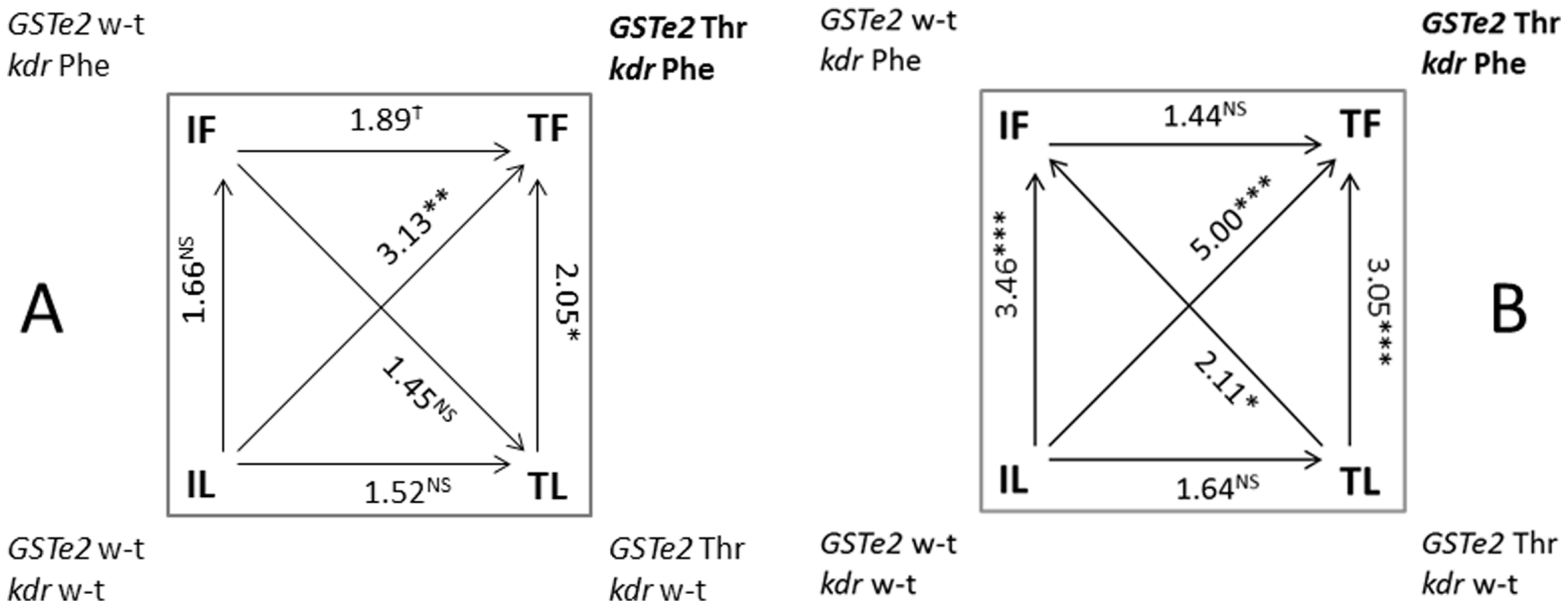
Summary of haplotypic association tests for the combination of four possible allele combinations at the *Vgsc*-1014 (*kdr*) and *Gste2*-114 loci with DDT susceptibility in An.gambiae M-form females from Benin (Panel A) and Burkina Faso (Panel B). Susceptibility to 4% DDT, was determined following a 1hr exposure to followed by 24hr recovery. Odds ratios are given with significance indicated by asterisks (? P = 0.0502,*P<0.05, **P<0.01, ***P<0.001). The arrow is oriented from least to most resistant. The allele combination in bold (*Gste2*-*114T*: *kdr*-*Phe*) is the double mutant which is significantly associated with DDT resistance. wt  =  wildtype.

For the Benin data, where both *Gste2*-114T and *Vgsc-1014F* were significantly associated with DDT resistance in univariate analyses, we fitted a general linear model with a logistic link function. In this analysis both mutations remained significantly associated with the ability of mosquitoes to survive DDT exposure (*Gste2*-114T p = 0.002: *Vgsc-1014F* p = 0.018). The additive effects of the resistance loci was revealed in both Benin and Burkina Faso by elevated odds ratio for a double mutant haplotype relative to wildtype (OR Benin = 3.13 (95% CIs 1.59–6.15; p = 0.0012: OR Burkina Faso 5.00 (95% CIs 2.51–9.98; p<<0.001: Figure 6; Datasets S1 and S2). The third mutation, *Vgsc*-1575Y, is at low frequency in Benin (Freq  = 0.035; 95%CIs 0.02–0.06) precluding association analysis but at a higher frequency in Burkina Faso (Freq  = 0.12; 95%CIs 0.09–0.16). In Burkina Faso *Vgsc-1014F* was strongly resistance-associated (p = 6.6×10^−7^) whereas both *Gste2*-114T (p = 0.051) and *Vgsc-1575Y* (p = 0.039) were on the margins of significance. However, for the triple mutant (*Gste2*-114T: *Vgsc-1014F* : *Vgsc*-1575Y) the odds ratio relative to wild type rose to 12.99 (95% CIs 2.55–66.10; p<0.001; Dataset S2), which translates into an increase in probability of surviving a one hour DDT exposure from 50% to 93%. Nonetheless, over 50% of the variation remained to be explained and may reflect the effects of environmental factors or additional resistance mechanisms (e.g.[8]).

Full-length *Gste2* sequences were obtained from 18 M-form individuals used in the Burkinabe genotype: phenotype tests (Genbank accession numbers: KC533009-KC533026). There were no additional non-synonymous mutations that segregated with the 114T mutation providing further evidence that mutation is causal, rather than merely a marker of DDT resistance.

## Discussion

Our data demonstrate how introgression of adaptively advantageous alleles between the molecular forms of *An. gambiae* can bring together combinations of alleles that enhance insecticide resistance phenotypes. This is yet another example of the evolutionary plasticity of this species complex and vividly illustrates why its members are so extremely difficult to control. The triple mutant described in this study is almost completely resistant to DDT, as assessed using the standard World Health Organization exposure assay. There is no simple association between resistance phenotype and epidemiological outcomes but these data raise concerns about the efficacy of indoor residual spraying with DDT in parts of West Africa for controlling malaria.

Insecticide resistance in mosquitoes [7], [9], [11], [12], [22], [23], and other insects [24], [25], is commonly linked to elevated expression of detoxifying enzymes. I*Nde*ed *Gste2* was first implicated in DDT resistance as a result of elevated expression rather than allelic variation [12], [13]. However, it seems that the ZAN/U strain used in earlier work bears little relation to that used in this study: in addition to the higher levels of *Gste2* expression observed, the amino acid at codon 114 was an asparagine (N) [12], [13], [26] not the threonine we identify here. The occurrence of the I114T mutant in our ZAN/U strain is probably a result of a contamination event, most likely from an M form colony, followed by selection during routine colony husbandry to maintain the DDT-resistant phenotype. Such inter-colony contamination events are a major problem when rearing morphologically identical mosquito strains[27]. The involvement of metabolic allelic variants in conferring an insecticide resistance phenotype is not without precedent. In the sheep blowfly, *Lucilia cuprina*, Newcomb et al. [1] highlighted a G137D substitution within a carboxylesterase gene, E3, which conferred broad-spectrum organophosphate (OP) hydrolase activity. The same mutation was subsequently found to confer OP resistance in the housefly *Musca domestica* [28]. Next generation sequencing of individual *An. gambiae* (http://www.malariagen.net/node/287) will permit genome-wide association studies of insecticide resistance phenotypes to simultaneously uncover coding and regulatory variants.

The data that were obtained from the heterologous expression of Kisumu and ZAN/U alleles in D. *melanogaster* are somewhat at odds with our contention that the ZAN/U allelic variant is DDT-resistance associated. However, these data may point to the influence of genotypic background in the penetrance of a resistance-associated variant, as has been observed previously in both *An. gambiae* and D. *melanogaster* [6], [30]. In an earlier study *Drosophila* transformed with the *Gste2*-ZAN/U allele showed DDT LC50 values in excess of those observed here [29].

### Mechanism of action of *Gste2-114T*

The importance of mutation I114T most likely arises from the creation of an enzyme with increased catalytic activity through predicted increased conformation dynamics and reduced product affinity, facilitating metabolic turnover. The relationship between structure, stability and catalysis of enzymes has been studied extensively in the context of protein thermostability [31]. Enzymes from hyperthermophiles, which grow optimally at elevated temperatures, are often barely active at room temperature but are as active as their mesophilic homologues at high temperatures. It has been proposed that the low activity of the thermostable enzymes at mesophilic temperatures is due to a high structural rigidity, which is relieved at their elevated physiological temperatures. This concept of “corresponding states” highlights the importance of protein dynamics in catalysis [32]. In agreement with this concept, rational protein design and directed evolution have shown that enzyme mutants with reduced stability often exhibit improved catalytic activity compared to the wild-type form, even though structural alterations are often minimal or u*Nde*tectable (e.g. [33], [34]). The lack of notable structural differences between the Kisumu 2B and ZAN/U 1C variants and the intrinsic dynamics of the region vicinal to the catalytic site in *GSTE2* enzymes led us to speculate an effect of the residue exchanges in protein stability. We predicted changes in stability that might result from mutation of amino acids, I114 and F120, to their smaller replacements, T114 in ZAN/U 1C and L120 in Kisumu 1B. The I114T change was predicted as strongly destabilising at 2.85 kcal/mol [35], while the F120L was classified as neutral at –0.98 kcal/mol. The destabilizing effect of the T114 exchange is likely due to the reduction in side chain volume, with the introduced polarity apparently well accommodated in the local environment. The change in volume is greater for position 120, but volume changes in protein cores are especially disruptive [36] and I114 is buried while F120 is largely solvent-accessible. It is position 114 that correlates better with activity and which was shown to associate with phenotype in the phenotypic work conducted in Benin and Burkina Faso (Figure 6). It appears that the 114 mutant drives DDT resistance through dynamic rather than static conformational changes.

## Conclusion

We describe a variant *Gste2*-114T that is significantly associated with DDT resistance in M molecular form females from West Africa. This mutation in concert with *Vgsc* mutations confers highly elevated resistance to DDT. Whilst individually the mutations may have a modest effect on resistance phenotype the effect of acquisition of these incremental changes relative to wild-type may be large.

## Materials and Methods

### Strains

The DDT resistant ZAN/U strain was derived from the ZANDS strain, colonised from Zanzibar and displaying resistance to DDT as larvae [10], [37]. ZAN/U was derived from this strain via selection of 1-day old adults with 4% DDT [38]. ZAN/U displays DDT resistance in the absence of known knockdown resistance (*kdr*) mutations in the sodium channel. The Kisumu strain is fully susceptible to DDT and originates from Kisumu in Western Kenya. Both of these laboratory colonies are of the S molecular form and originate from East Africa. These studies did not involve human participants or endangered or protected species and therefore no ethical clearance of specific permissions were required.

### Sequencing of *Gste2*

*Gste2* (GenBank accession number XM319968.3), for which only a single transcript has been reported, is situated on chromosome 3R at position 28,597,686–28,598,594 (AgamP3.5 genome assembly of *An. gambiae* see www.vectorbase.org). To investigate non-synonymous changes between the strains, sequence data were obtained from ten individual female mosquitoes from both ZAN/U and Kisumu. Primers were designed to amplify a 680bp fragment encompassing the majority of the three exons. Total DNA was purified from single insects using the DNeasy Blood and Tissue spin column kit (Qiagen). All twenty DNA extracts were confirmed as the S-form of *An. gambiae* using a PCR-RFLP approach [39]. *Gste2* amplicons were sent for direct sequencing (Macrogen, South Korea). Those individuals yielding poor quality data from direct sequencing were re-amplified and cloned in Escherichia coli using a pGEM-T Easy Vector (Promega) prior to sequencing. All sequences were aligned versus the full *Gste2* genomic sequence obtained from VectorBase (http://www.vectorbase.org/) using CodonCode Aligner software (CodonCode Corporation) and synonymous and non-synonymous polymorphisms identified.

Full-length cDNA sequences for Kisumu and ZAN/U *Gste2* were produced from RNA extracted from three batches of ten female mosquitoes from each strain using the PicoPure RNA Isolation Kit (Arcturus). RNA concentration was measured (NanoDrop spectrophotometer, Thermo) and approximately 2 μg from each pool used for cDNA synthesis (SuperScript III Reverse Transcriptase, Invitrogen). The cDNA sequence was amplified from cDNA pools using primers situated in the 5’ and 3’ untranslated regions (Table S1 in File S1) to produce a 683bp fragment. The amplified *Gste2* fragment from each cDNA pool was then cloned into a pGEM-T Easy holding vector (Promega) using 1 μl of PCR product. Positive clones from each cDNA pool were selected for sequencing. Selected clones were used to inoculate a 5ml, over-night culture from which plasmid DNA was extracted (QIAprep Spin Miniprep Kit, Qiagen). An aliquot of each plasmid was then sent for sequencing (Macrogen, South Korea; GenBank accession numbers: JX840597- JX840599).

### Modelling of non-synonymous changes on to the GSTE2 protein structure

The amino acid changes identified in the ZAN/U and Kisumu sequence data were interpreted in the context of the Kisumu *GSTE2* crystal structure [ProteinDataBank (PDB) accession code 2IL3] and their potential importance in DDT binding inferred with respect to the residues highlighted by Wang et al. [15] as amino acid positions likely to be involved with DDT binding/metabolism, henceforth termed the catalytic triad. This *in silico* approach was used to select *Gste2* haplotypes that were likely to have differing DDT-ase activity for further recombinant protein and crystallography work. PoPMuSiC [35], [36] was used to predict protein stability changes occurring as a result of amino acid changes between the polymorphisms.

### Recombinant protein expression and DDTase activity screens

Recombinant protein expression was performed for three *Gste2* allelic variants that had non-synonymous changes proximal to the DDT binding site. *Gste2* was re-amplified from clones of the cDNA extracts using primers that incorporated a 3’*Nde*I site and a 5’ *Bam*HI restriction site (Table S1 in File S1). These restriction sites were exploited to clone the *Gste2* alleles into protein expression vector pET15b (Novagen) before transformation into *E. coli* BL21 (DE3) (New England Biolabs). Cultures were incubated at 37°C (150RPM) until an optical density of ≈0.8 (wavelength 600 nm) was reached, then protein production was induced by addition of 1 mM isopropy-β-D-thiogalactoside (IPTG) at 30°C (150RPM). A pET15b encoded polyhistidine (6XHis) tag was exploited for purification of GSTE2 using nickel affinity chromatography. Bacterial lysates were prepared by sonication in buffer TSE (50 mM Tris-HCl pH 7.4, 1 mM EDTA, 150 mM NaCl, 10 mM β-mercaptoethanol (β -ME), 1.25 mM MgCl2 and 250 U benzonase) and cell debris removed through centrifugation (10,000 g for 20 minutes at 4°C) and filtration (0.2 μm filter). Crude cell lysate was then applied to a 1 ml nickel- nitrilotriacetate (Ni-NTA) agarose (Qiagen) column and washed with 10 column volumes of buffer A (50 mM sodium phosphate, 200 mM NaCl, pH 8.0) containing 20 mM imidazole. Protein was eluted in 10 ml of buffer B (50 mM sodium phosphate, 300 mM NaCl, pH 8.0) containing 250 mM imidazole. Purified *GSTE2* was then applied to a PD-10 Desalting Column (GE Healthcare) and eluted in storage buffer [50 mM sodium phosphate, 20 mM Dithiothreitol (DTT), pH 7.4].

Protein concentration was determined using a commercial protein quantification kit (Fluka – Sigma-Aldrich) based on the Bradford protein assay [40] and GST activity confirmed for each purified recombinant variant using the GST substrate 1-chloro-2, 4-dinitrobenzene (CDNB) in a standard colorimetric activity assay [41]. The recombinant proteins produced for each of the three *GSTE2* variants were of extremely high and consistent purity (Figure S4 in File S1).

The DDT dehydrochlorinase activity of all *GSTE2* variants was assessed using an enzymatic assay and High Performance Liquid Chromatography [10]. *GSTE2* catalyses the dehydrochlorination of DDT in the presence of glutathione (GSH) to produce 1,1-dichloro-2,2-bis(p-chlorophenyl) ethylene (DDE)[12]. Reverse-phase HPLC using a silica based stationary phase and a 90%:10% methanol:water mobile phase was used to separate DDT, DDE and the spike-in control dicofol according to their polarity. Standard curves were produced for DDT, DDE and dicofol using a doubling dilution series (200 – 12.5 μg/ml). The mobile solvent phase was pumped through the HPLC system (Ultimate 3000) at a rate of 1 ml/minute and 20 μl of each sample injected. Data acquisition was set at 18 minutes as DDE elutes at approximately 14 minutes with DDT eluting at ≈12 minutes, and the UV wavelength 232 nm selected. Compound concentration (μg/ml) was then plotted against the HPLC peak area to produce a standard curve with the intercept fixed at zero. The equation of this curve was employed to assess DDT, DDE and dicofol concentration in subsequent assays.

### Enzyme kinetics

To compare enzyme activity between variants, a doubling dilution series of DDT from 200–3.125 μg/ml was employed using optimised reaction parameters. Each assay contained 60 μg of *GSTE2* enzyme. All variant *GSTE2* proteins were assayed at each DDT concentration and a series of three technical replicates performed. The DDE peak area from the HPLC trace was normalised against the dicofol spike-in area and the adjusted area used to calculate micrograms of DDE produced per ml reaction using the DDE standard curve. The DDE concentration was used to calculate the enzyme rate, expressed as μmol DDE/mg GSTE2 protein/min. Michaelis-Menten and substrate inhibition plots were produced to compare the kinetics of each GSTE2 allele based upon initial substrate concentration (DDT) and rate of product (DDE) formation in R [42]. The maximum enzyme rate (V_max_), the point at which all enzyme active sites are bound to substrate, the Michaelis-Menten constant (K_M_), which is the substrate concentration for an enzyme at half its maximum velocity and K_cat_, a measurement of overall catalytic turn-over rate, were derived from the fitted equations.

### X-ray crystallography and corresponding recombinant protein production

The *Gste2* variant ZAN/U was cloned into the expression vector pOPIN (Oxford Protein Production Facility-UK) via the In-Fusion PCR cloning system (Clontech). This vector incorporates His_6_- and SUMO-tags, as well as a SUMO protease cleavage site, N-terminal to the target insert. Protein expression was in *E. coli* BL21(*DE3*) Rosetta2 (Novagen). Cultures were grown at 37°C up to an OD_600_ of 0.6 in Terrific broth supplemented with 50 μg/ml kanamycin and 34 μg/ml chloramphenicol. Expression was induced with 1 mM IPTG and cultures grown for a further 18hr at 25°C. Cells were harvested by centrifugation. The bacterial pellet was re-suspe*Nde*d in lysis buffer (25 mM Tris-HCl pH 8.0, 500 mM NaCl, 5 mM β-ME) and supplemented with 1.25 mM MgCl_2_ and 250 units of benzonase before sonication on ice. The homogenate was clarified by centrifugation and affinity purified using a 3 ml Ni-NTA agarose (Qiagen) column equilibrated in wash buffer (lysis buffer supplemented with 20 mM imidazole). Protein was eluted using 250 mM imidazole before over-night dialysis at 4°C against 25 mM Tris-HCl pH 8.0, 200 mM NaCl, 5 mM β-ME to remove imidazole and reduce salt concentration. Tags were removed by incubation with SUMO protease overnight at 4°C (1.7 μl SUMO protease/mg fusion protein). Further purification used subtractive metal affinity and size exclusion chromatography in a Superdex 75 HR16/60 column (GE Healthcare) equilibrated in dialysis buffer. Purified samples were concentrated to 13 mg/ml via Vivaspin column (GE Healthcare). As the apo enzyme was unstable and degraded rapidly, it was supplemented with GSH (1∶1.2 molar ratio) and the stabilized complex stored at 4°C until further use.

Crystals of ZAN/U:GSH were grown at 22°C in VDX 24-well plates in hanging drops. Drops consisted of 1 μl protein solution and 1 μl mother liquor containing 30% PEG 6000, 0.1 M Bis-Tris pH 6.5, 1 mM β-ME. Crystals grew within 3 days and exhibited rod morphologies with approximate dimensions of 0.2×0.05×0.05 mm^3^. Crystals were then soaked in mother liquor supplemented with 40% PEG 400 and DDE at saturation for 2 days. For X-ray data collection, crystals were retrieved and shock-frozen in liquid nitrogen. Diffraction data were collected at 100 K on beamline I04 at Diamond (Didcot, UK) and processed using XDS/XSCALE [43]. Processing statistics and crystallographic parameters are given in Table S2 in File S1. The crystal form used in this study contained two biological dimers in its asymmetric unit (four molecular copies). Phasing was by molecular replacement in Phaser[44] using a single molecular copy (A) from PDB entry 2IL3 [15]. The model was manually rebuilt in COOT [45] and TLS refined in Refmac5 using local NCS restraints [46]. Solvent building was in Phenix [47] and COOT. In the final model, the four molecular copies of ZAN/U:GSH were virtually identical (0.42 Å overall rmsd calculated with RAPIDO [19]). DDE binding could not be identified in electron density maps. Model and refinement statistics are given in Table S2 in File S1. Model coordinates and diffraction data have been deposited with the ProteinDataBank (PDB accession code 4GSN).

### Heterologous expression of GSTE2 in *Drosophila melanogaster*

cDNA clones including the open reading frames for *Gste2*-ZAN/U and *Gste2*-Kisumu1B, were PCR-amplified using high fidelity *AccuPrime Pfx* polymerase (Invitrogen). The PCR primers used contained *Eco*RI and *Not*I restriction sites within the forward and reverse primers, respectively (Table S1 in File S1). PCR products were gel-purified using the GenElute Gel Extraction Kit (Sigma) and subsequently ligated into a pUASTattB plasmid (obtained from Dr. Konrad Basler, University of Zurich) using T4 DNA ligase (New England Biolabs). Ligation mixtures were transformed into competent DH5α cells for plasmid production, and individual colonies were verified using PCR. The EndoFree Plasmid Maxi Kit (Qiagen) was utilized to obtain purified plasmid DNA for subsequent steps. pUAST-attB clones containing *Gste2* inserts were sent to Rainbow Transgenic Flies, Inc. (Camarillo, CA, USA) for injection into Bloomington stock #9750 (y^1^ w^1118^; PBac{y^+^-attP-3B}VK00033) embryos. This Phi integration system enables site-specific recombination between the integration vector (pUAST-attB) and a landing platform in the fly stock (attP)[48].

Larvae were kept at 25°C, and G_o_ flies that eclosed were sorted by sex prior to mating. To establish families of homozygous transgenic flies, G_o_ flies were crossed with w^1118^ flies and G_1_ flies were sorted based on w^+^ eye color (as a marker for insertion events). G*_1_* w^+^ flies were crossed *inter se* to obtain homozygous insertion lines. The following *D. melanogaster* stocks were obtained from the Bloomington *Drosophila* Stock Center (Bloomington, IN, USA): y^1^ w^1^; P{Act5C-GAL4}25FO1/CyO, y^+^ and w^1118^ (BL3605). Virgin females from both types of *Gste2* insertion stocks were crossed with *Act5C-GAL4/CyO* (ubiquitous Actin5C driver) flies. Control crosses were set up in parallel by crossing heterozygous (*Act5C*) GAL4 driver males to virgin *w^1118^* females.

To create dose response curves *Drosophila* adults were exposed to a range of DDT concentrations (Figure 4). DDT, dissolved in 100 μl of acetone, was added to 16×100 mm glass disposable culture tubes (VWR Scientific). Tubes were placed on their sides and continually oscillated until the entirety of the interior of tube was coated and all acetone had evaporated. A total of 8–12 control and 8–12 experimental transgenic flies were added to each tube. The tubes were capped with cotton wool saturated with a 10% (w/v) glucose/water solution. Tubes were then incubated at 25°C for 24hr. After 24hr, mortality, (as indicated by absence of movement) was recorded and LC_50_ values calculated in the R language [42].

### Screening of allelic variants in wild-caught, DDT-phenotyped specimens of *Anopheles gambiae*

Data from catalytic assays, modelling and X-ray crystallography suggested that one of the non-synonymous changes had a marked effect on DDTase activity. A TaqMan SNP genotyping assay was designed to screen for the mutation in individual mosquitoes (see Table S1 in File S1 for primer and probe sequences). DNA extracts from adult female mosquitoes from a number of locations in sub-Saharan Africa were genotyped for the *Gste2* allelic variants. In addition female mosquitoes with known DDT susceptibility phenotypes, as defined by the standard WHO protocol, were obtained from Burkina Faso[49] and Benin. SNP genotyping assays were performed in 10 μl volume containing 1x Sensimix (Bioline), 1x primer/probe mix and 1 μl template DNA with a temperature profile of 95°C for 10min followed by 40 cycles of 92°C for 15s and 60°C for 1min on an Agilent MX3005 real-time PCR machine. VIC and FAM fluorescence was captured at the end of each cycle and genotypes called from endpoint fluorescence using the Agilent MXPro software. Specimens from Benin and Burkina Faso were also screened for known DDT-resistance associated variants in the voltage-gated sodium channel [5], [50]. Genotype: phenotype associations were assessed using a generalized linear model with a logit link function [42], chi-squared tests Poptools 3.2 [51], and sample haplotype frequencies estimated using Haploview 4.2 [52].

## Supporting Information

File S1 **This file contains Figure S1-S4 and Table S1-S3.** Figure S1, Mean normalised expression of *GSTe2* in female *An. gambiae* s.s. of the DDT resistant ZAN/U strain and susceptible Kisumu strain. Expression of *GSTe2* and ribosomal S7 were assessed from ten RNA pools comprised of ten 3 day old female mosquitoes using the GeXP quantitative PCR system (Beckman-Coulter). The ZAN/U colony showed 2.34 fold greater expression of *Gste2* compared with the Kisumu colony. *GSTe2* expression was normalised against housekeeping gene ribosomal S7. Standard error of the normalised mean expression is also indicated. Figure S2, Western blot comparison of *GSTe2* protein level in the Kisumu (Kis) and ZAN/U (Zan) *An. gambiae* s.s. strains. Whole mosquito extracts from 10 unmated 3 day old female mosquitoes from each strain was probed with *An. gambiae GSTe2* polyclonal antibody. Approximately 1.7 times more *GSTe2* protein was present in the ZAN/U extract as determined by background corrected pixel intensities using the ImageJ v1.43 software. Ae. aegypti recombinant *GSTe2* was run as a positive control (+). Figure S3, Superimposition of the *GSTE2* enzymes of *An. gambiae* (ZAN/U variant generated in this study containing Thr114; orange) and *An. funestus* (containing Ile114; blue). The GSH ligand is shown in red. a. Overall view; b. close-up of the mutated region of helix H4 showing the altered residue in position 114, and Phe113 at the dimer interface. Figure S4, SDS PAGE gel illustrating the purity of three recombinant variants of *Gste2* isolated from *An. gambiae* s.s. The left panel represents 2.5 μg and the right 1.25 μg of each glycerol stored protein. SDS PAGE performed as previously outlined. Table S1, PCR primers used in the study. Numbers 1 and 2 - *Gste2* promoter region amplification and sequencing. Numbers 3 and 4-amplification of the *Gste2* coding region. Numbers 5 and 6- amplification of the coding region of *Gste2* incorporating the *NdeI* and *BamHI* restriction enzyme sites for subsequent cloning into expression vector pET-15b (Novagen). Numbers 7 and 8 Heterologous expression of *GSTE2* in *Drosophila melanogaster*. Numbers 9–12 primers and probes used in the Taqman assay for variants at the 114 codon. Probes 11 and 12 carried a non-fluorescent quencher at the 3’end. Table S2, Statistics for X-ray data and model refinement. The model contains all protein residues with the exception of Ala221 in chain A and C and Lys220-Ala221 in chain D that were disordered in the electron density maps. Table S3, Showing exact collection latitudes and longitudes of the collections used in figure 5.
(DOCX)

Dataset S1 (CSV)

Dataset S2 (CSV)
