# Supplementary material for: Metabolic and Target-Site Mechanisms Combine to Confer Strong DDT Resistance in Anopheles gambiae
Source: PLoS One. 2014 Mar 27;9(3):e92662. doi: 10.1371/journal.pone.0092662 (PMC3968025; doi:10.1371/journal.pone.0092662)
Supplement: File S1 — This file contains Figure S1-S4 and Table S1-S3. Figure S1, Mean normalised expression of GSTe2 in female An. gambiae s.s. of the DDT resistant ZAN/U strain and susceptible Kisumu strain. Expression of GSTe2 and ribosomal S7 were assessed from ten RNA pools comprised of ten 3 day old female mosquitoes using the GeXP quantitative PCR system (Beckman-Coulter). The ZAN/U colony showed 2.34 fold greater expression of Gste2 compared with the Kisumu colony. GSTe2 expression was normalised against housekeeping gene ribosomal S7. Standard error of the normalised mean expression is also indicated. Figure S2, Western blot comparison of GSTe2 protein level in the Kisumu (Kis) and ZAN/U (Zan) An. gambiae s.s. strains. Whole mosquito extracts from 10 unmated 3 day old female mosquitoes from each strain was probed with An. gambiae GSTe2 polyclonal antibody. Approximately 1.7 times more GSTe2 protein was present in the ZAN/U extract as determined by background corrected pixel intensities using the ImageJ v1.43 software. Ae. aegypti recombinant GSTe2 was run as a positive control (+). Figure S3, Superimposition of the GSTE2 enzymes of An. gambiae (ZAN/U variant generated in this study containing Thr114; orange) and An. funestus (containing Ile114; blue). The GSH ligand is shown in red. a. Overall view; b. close-up of the mutated region of helix H4 showing the altered residue in position 114, and Phe113 at the dimer interface. Figure S4, SDS PAGE gel illustrating the purity of three recombinant variants of Gste2 isolated from An. gambiae s.s. The left panel represents 2.5 μg and the right 1.25 μg of each glycerol stored protein. SDS PAGE performed as previously outlined. Table S1, PCR primers used in the study. Numbers 1 and 2 - Gste2 promoter region amplification and sequencing. Numbers 3 and 4-amplification of the Gste2 coding region. Numbers 5 and 6- amplification of the coding region of Gste2 incorporating the NdeI and BamHI restriction enzyme sites for subsequent cloning into e [file pone.0092662.s001.docx]

**Supplementary table 1.**

|  | Primer/ probe name | Primer/ probe sequence |
| --- | --- | --- |
| 1 | AGU1 F | 5'-TTGCCGTACTATGAGGAGATCAAC-3' |
| 2 | AGU1 R | 5'-TCTCTCTCAATCCCTTTACGTACC-3' |
| 3 | GSTe2 cDNA F | 5’-CGCTGCGAAAATGTCCAACC-3’ |
| 4 | GSTe2 cDNA Rb | 5’-TACCTTTTTAAGCCTAGCATTC-3’ |
| 5 | GSTe2 cDNA_RE_F | ***NdeI***  5’-TTT***CATATG***TCCAACCTTGTAC-3’ |
| 6 | GSTe2 cDNA_RE_R | ***BamHI***  5’-TTT***GGATCC***TAAGCCTTAGCATTC-3’ |
| 7 | GSTe2_*fEcoRI* | 5’-GAATTCATGTCCAACCTTGTACTGTACACC-3’ |
| 8 | GSTe2_*rNotI*: | 5’-GCGGCCGCTTAAGCCTTAGCATTCTCCTCCTT -3’ |
| 9 | 114-Taqman primer F | 5’-CGAGTCCGGCGTACTGTT-3’ |
| 10 | 114-Taqman primer R | 5’-GGCGTTATGCTGGAACTGGAA |
| 11 | 114 Taqman probe ILE | 5’-6FAM-ACGAAAATGAATCTC-3’ |
| 12 | 114 Taqman probe THR | 5’-VIC-ACGAAAGTGAATCTC-3’ |

**Supplementary table 2.**

*Diffraction data*

X-ray source I04, Diamond (Didcot)

Detector ADSC Quantum 315

Wavelength (Å) 0.9763

Spacegroup P2_1_

Unit cell a=51.33 Å, b=86.38 Å, c=92.85 Å, β=90.73

Molecules a.u. / solvent content 4 / 40%

Resolution 20.0-2.3 (2.35-2.30)

Unique reflections 35941 (2245)

R_sym_ (I) [%] 10.4 (50.7)

I/σ (I) 10.4 (3.3)

Multiplicity 3.72 (3.82)

Completeness (%) 99.4 (99.2)

*Refinement statistics*

Nr Reflections in working / test sets 34800 / 1123

R‑factor/R_free_ (%) 17.57 / 22.78

Nr protein residues^a^ / ligands / solvent atoms 878 / 4 x GSH / 198

Total number of atoms 7260

Average B-factors (Å^2^) 38.0

rmsd bond / angle (^o^) 0.002 / 0.636

Ramachandran analysis

Favoured / Allowed / Outlier (%) 97.7 / 1.8 / 0.5

**Supplementary table 3.**

| **Country** | **Location name/comment** | **Latitude** | **Longitude** |
| --- | --- | --- | --- |
| Benin | Cotonou | 06°21'45”N | 02°25’32”E |
| Benin | Pahou | 06°22'60”N | 02°13’00”E |
| Benin | Tori-Bossito | 06°30'11”N | 02°08'42”E |
| Benin | Bohicon | 07°10'08”N | 02°04’01”E |
| Benin | Sekou | 06°37'00”N | 02°13'00”E |
| Benin | Glazoue | 07°58'25”N | 02°14'24”E |
| Benin | Kandi | 11°07' 43”N | 02°56'13”E |
| Benin | Malanville | 11°52'00”N | 03°22’60”E |
| Burkina Faso | Soumosso | 11° 01’ 12”N | 04° 03’ 00”W |
| Burkina Faso | Goundry | 12° 30’ 00”N | 01° 20’ 00”W |
| Burkina Faso | Koupela | 12° 11’ 50”N | 00° 21’ 21”W |
| Burkina Faso | Kuinima | 11° 08’ 49”N | 04° 17’ 00’’W |
| Cameroon |  | 03° 52’ 00’’N | 11° 31’ 0”E |
| Ghana |  | 05° 53’ 00’’N | 00°00’ 00’’W |
| Guinea Bissau |  | 11° 53’ 28”N | 15° 34’ 55’’W |
| Mali |  | 13° 24’ 00”N | 7° 7’ 48”W |
| Uganda |  | 00° 41’ 34’’N | 34° 10’ 52”E |

**Supplementary figure 1.**

**Supplementary figure 2.**

**

**Supplementary figure 3.**


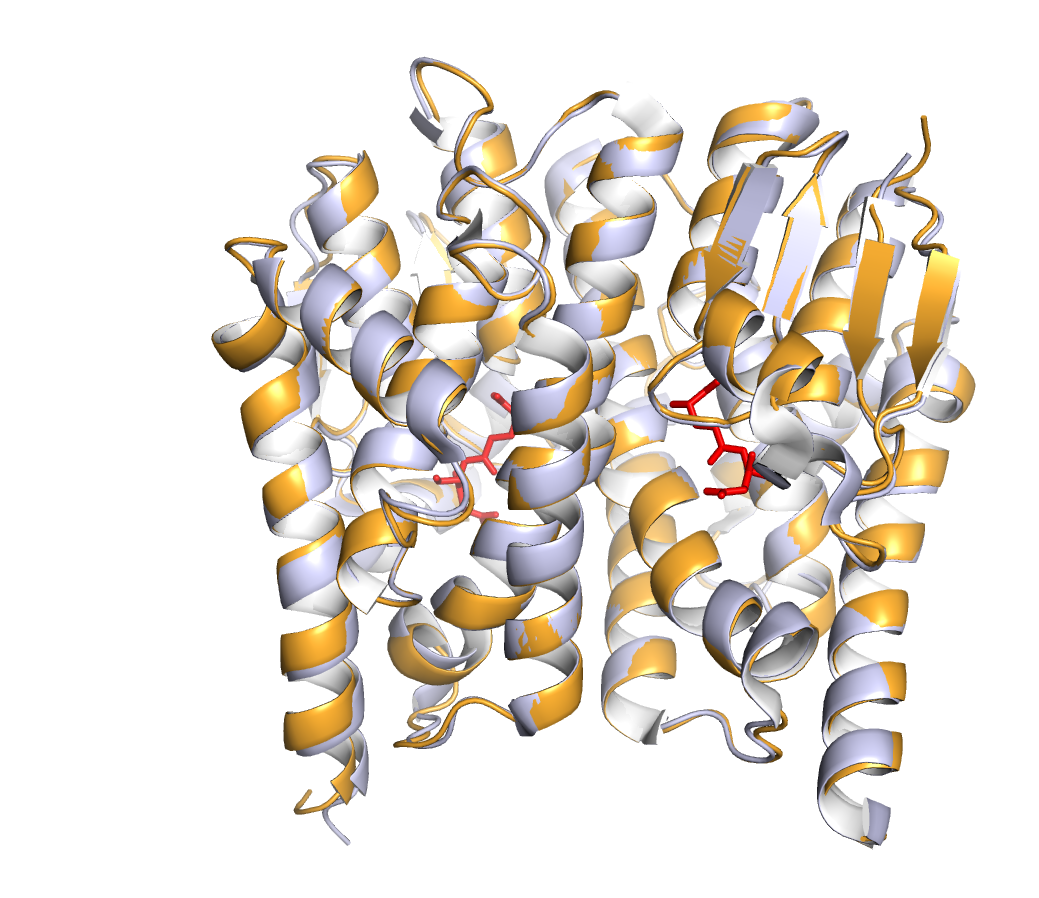


**a. b.**


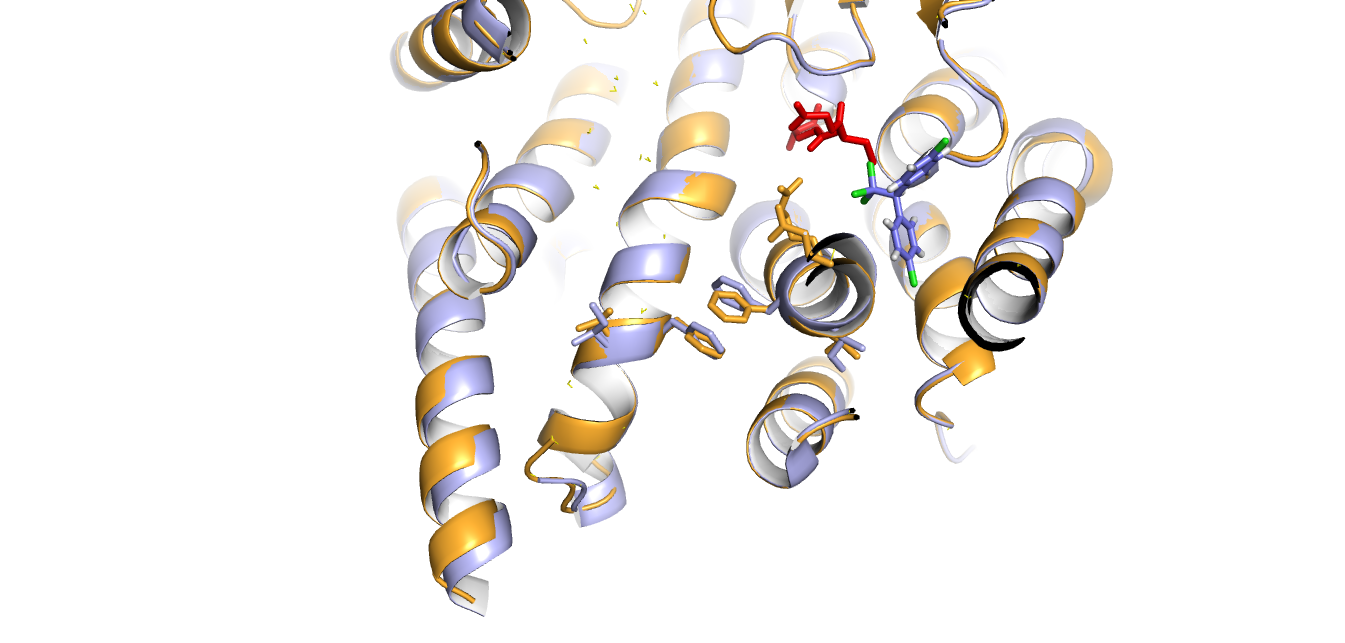


**H4’**

**113’**

**114**

**H4**

**113**

**114’**

**Supplementary figure 3**


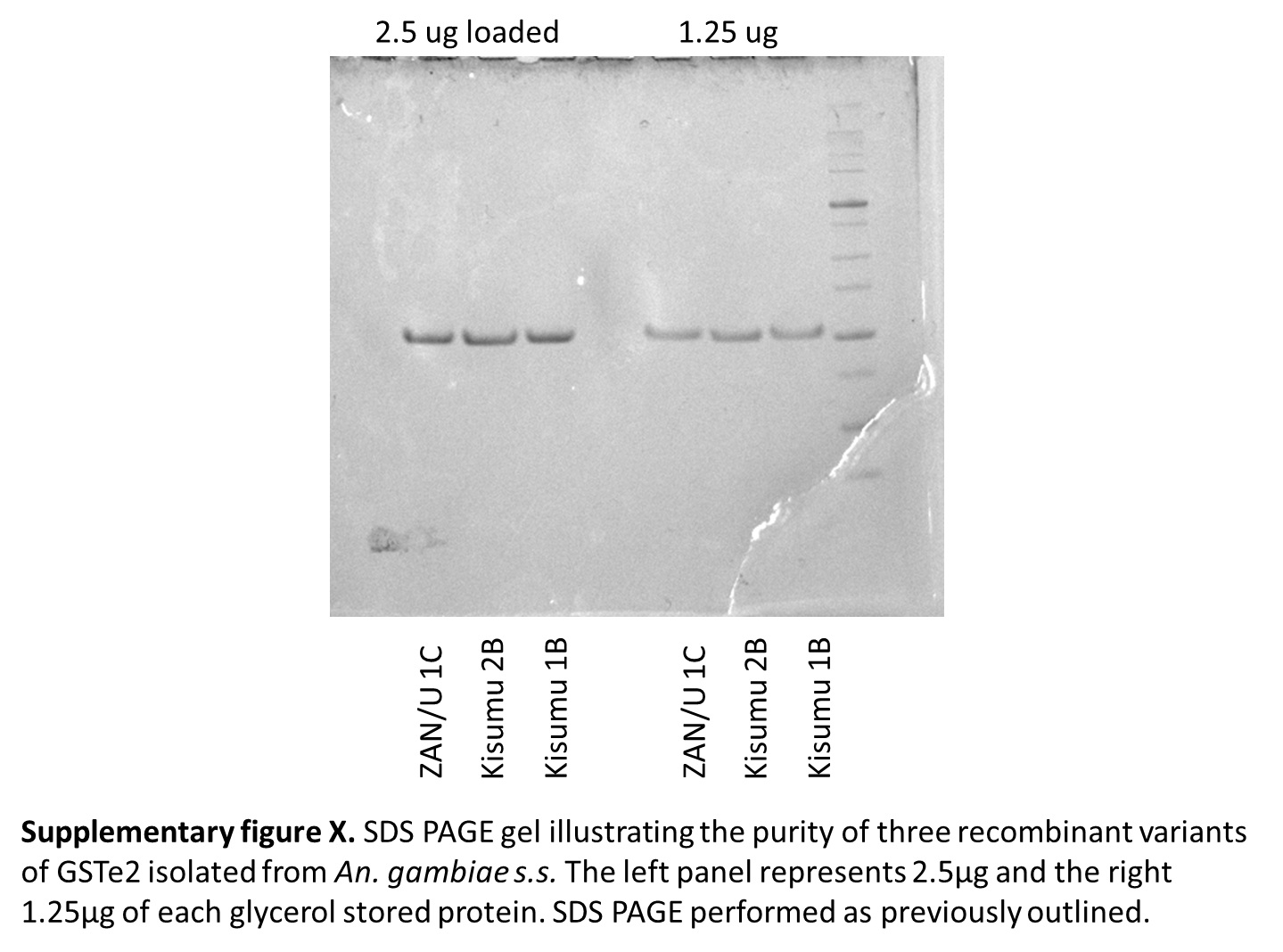
 **Supplementary figure 4**
